# 3D Printing Type 1 Bovine Collagen Scaffolds for Tissue Engineering Applications—Physicochemical Characterization and In Vitro Evaluation

**DOI:** 10.3390/gels9080637

**Published:** 2023-08-08

**Authors:** Vasudev Vivekanand Nayak, Nick Tovar, Doha Khan, Angel Cabrera Pereira, Dindo Q. Mijares, Marcus Weck, Alejandro Durand, James E. Smay, Andrea Torroni, Paulo G. Coelho, Lukasz Witek

**Affiliations:** 1 Department of Biochemistry and Molecular Biology, University of Miami Miller School of Medicine, Miami, FL 33136, USA; vxn188@miami.edu (V.V.N.); pgc51@med.miami.edu (P.G.C.); 2 Biomaterials Division, NYU College of Dentistry, New York, NY 10010, USA; nicktovar@gmail.com (N.T.); dk3594@nyu.edu (D.K.); ac8849@nyu.edu (A.C.P.); dqm1@nyu.edu (D.Q.M.); 3 Department of Oral and Maxillofacial Surgery, New York University, Langone Medical Center and Bellevue Hospital Center, New York, NY 10016, USA; 4 Department of Chemistry and Molecular Design Institute, New York University, New York, NY 10003, USA; mw125@nyu.edu; 5 Department of Biomedical Engineering, NYU Tandon School of Engineering, Brooklyn, NY 11201, USA; ajd9371@nyu.edu; 6 School of Materials Science and Engineering, Oklahoma State University, Tulsa, OK 74106, USA; smay@okstate.edu; 7 Hansjörg Wyss Department of Plastic Surgery, NYU Grossman School of Medicine, New York, NY 10016, USA; andrea.torroni@nyumc.org; 8 DeWitt Daughtry Family Department of Surgery, Division of Plastic Surgery, University of Miami Miller School of Medicine, Miami, FL 33136, USA

**Keywords:** bovine collagen, crosslinking, lyophilizing, tissue engineering, additive manufacturing, 3D printing

## Abstract

Collagen, an abundant extracellular matrix protein, has shown hemostatic, chemotactic, and cell adhesive characteristics, making it an attractive choice for the fabrication of tissue engineering scaffolds. The aim of this study was to synthesize a fibrillar colloidal gel from Type 1 bovine collagen, as well as three dimensionally (3D) print scaffolds with engineered pore architectures. 3D-printed scaffolds were also subjected to post-processing through chemical crosslinking (in N-(3-Dimethylaminopropyl)-N′-ethylcarbodiimide) and lyophilization. The scaffolds were physicochemically characterized through Fourier Transform Infrared Spectroscopy (FTIR), Thermogravimetric Analysis, Differential Scanning Calorimetry, and mechanical (tensile) testing. In vitro experiments using Presto Blue and Alkaline Phosphatase assays were conducted to assess cellular viability and the scaffolds’ ability to promote cellular proliferation and differentiation. Rheological analysis indicated shear thinning capabilities in the collagen gels. Crosslinked and lyophilized 3D-printed scaffolds were thermally stable at 37 °C and did not show signs of denaturation, although crosslinking resulted in poor mechanical strength. PB and ALP assays showed no signs of cytotoxicity as a result of crosslinking. Fibrillar collagen was successfully formulated into a colloidal gel for extrusion through a direct inkjet writing printer. 3D-printed scaffolds promoted cellular attachment and proliferation, making them a promising material for customized, patient-specific tissue regenerative applications.

## 1. Introduction

Tissue engineering strategies focus on the regeneration and restoration of the form and function of damaged tissue at the host site [1]. In recent years, research in this domain has pivoted towards the synthesis and evaluation of novel biomaterials for use as scaffolds [2]. Scaffolds for tissue repair have been fabricated using a plethora of materials such as glycosaminoglycans to mimic the extracellular matrix (ECM) within tissue [3,4,5]. However, the ECM mainly consists of collagen, a naturally occurring biocompatible polymer abundantly found in hard and soft tissues, making it a favorable choice for the synthesis of biomedical devices [6,7]. As the most abundant ECM protein, collagen regulates cell adhesion, proliferation, and differentiation [8].

Collagen possesses excellent biocompatibility, biodegradability, and low immunogenicity, making it a widely utilized biomaterial for medical applications and for the development of tissue engineering constructs such as resorbable scaffolds [9,10,11]. It is characterized by a hierarchical composition of microfibrils and fibers which form a right-handed triple helix [11,12], rendering it chemically tailorable for a variety of applications such as creating three-dimensional environments for the culture and differentiation of cells, and scaffolds for tissue regrowth [13,14]. The literature pertaining to collagen scaffolds has indicated numerous studies detailing their successful use in preclinical settings, albeit in smaller animal models [15,16].

However, complete regeneration of critically sized hard and soft tissue defects in larger animal models or more complex clinical cases necessitates the use of high aspect ratio, porous, spanning structures that permit the flow of blood and nutrients while acting as a conduit for tissue ingrowth [1,17]. Such intricate, geometrically complex, and large constructs warrant the use of innovative techniques for rapid prototyping of customized, patient, and anatomic location-specific scaffolds. The field of tissue engineering has hence seen tremendous progress in the use of additive manufacturing or 3D printing for producing various biomaterials. Importantly, the Direct Inkjet Writing (DIW) 3D printing technique for the fabrication of tissue engineering scaffolds for critically sized defects has been studied extensively [18,19]. Along with the ability to precisely control the overall shape of the scaffolds, the DIW technique has been shown to allow for the incorporation of engineered pore architectures with good repeatability and high precision [20]. However, the ideal ink or gel formulation for extrusion through a DIW printer should satisfy appropriate viscoelastic behavior [21]. Thus far, non-denatured collagen-based materials have not shown great potential for DIW directly onto a dry substrate due to two main reasons.

First, collagen remains in a liquid state at ambient temperatures. Low-viscosity collagen solutions render direct 3D printing on a dry substrate difficult to achieve [22]. As such, collagen scaffolds have been 3D-printed through cryogenic routes or in situ crosslinking [23,24]. Several studies have also reported methods that improve the collagen-based bioinks’ printability such as through a combination of hybrid collagen and synthetic polymers, ceramics, or printing collagen into a sacrificial support gel [25,26]. Second, collagen scaffolds printed through the aforementioned routes have been reported to present poor structural stability when used as tissue engineering constructs. Most commonly utilized methods of physical cross-linking, such UV irradiation, have been shown to improve mechanical strength and preserve biocompatibility [27]. However, UV irradiation has also been demonstrated to induce conformational changes in the triple helix structure of collagen in association with the chances of causing denaturation of collagen at higher intensities [27].

Therefore, chemical agents have been explored to improve the handling and use of collagen scaffolds through intermolecular cross-linking. Glutaraldehyde (GA), a synthetic cross-linking agent, has been widely used in crosslinking collagen-based constructs [28]. However, aldehydes—the functional groups of GA—are cytotoxic and cause inflammation [29]. In addition, GA could also induce undesirable calcification of the collagen-based constructs after implantation [30,31]. On the other hand, 1-ethyl-3-(3-dimethylaminopropyl)-carbodiimide hydrochloride (EDC), a covalent, zero-length cross-linking agent, has not been reported to cause any cytotoxic reactions [32,33]. However, information pertaining to the application of EDC in the crosslinking of 3D-printed fibrillar collagen scaffolds is lacking. In the present study, our group focused on the synthesis of a colloidal gel of fibrillar collagen with an appropriate viscoelastic response for direct extrusion onto a dry substrate using DIW for the rapid production of tissue engineering scaffolds. This work also aims to highlight the effect of EDC crosslinking on the mechanical and biological properties of the 3D-printed constructs. 

## 2. Results and Discussion

The versatility of collagen to serve as a tissue engineering scaffold can be attributed to its chemical similarity to the ECM of bone and soft tissue. More importantly, this key similarity has been indicated to be an essential factor in rendering collagen as a biomimetic material in the synthesis of tissue engineering scaffolds [34]. In this body of work, DIW printing was used to successfully fabricate collagen scaffolds by direct deposition of non-denatured, fibrillar collagen onto a dry substrate at room temperature. Furthermore, physicochemical characterization and in vitro analyses demonstrated the biological advantages of collagen scaffolds generated through additive techniques. 

In recent years, DIW has gained popularity in the tissue engineering domain as it allows for the extrusion of temperature-sensitive materials and for the incorporation of drugs and bioactive molecules directly into the feedstock material [35]. However, the rheological properties of the feedstock (colloidal gel or ink) limit the types of materials that can be printed [21]. In our work, a colloidal gel was synthesized by lyophilization of low-viscosity Type 1 bovine collagen and mixed with acetic acid (pH 2.4) to induce swelling of collagen fibers and fibrils by mechanical grinding and acidic regulation. This was primarily performed to achieve a high viscosity, glassy, fibrillar gel dispersion to facilitate extrudability and successive shape retention post-extrusion. At the molecular level, the formation of fibrillar collagen gel has been detailed to be a multi-step process [36]. The literature pertaining to fibrillar collagen synthesis describes the initial phase to include the association of collagen fibrils to form the nucleus of a triple helix structure, which then proceeds to form dimers or trimers of collagen and rearranges to form triple helices [37]. After a critical number of nuclei are formed, fibrils are described to grow laterally to yield collagen fibers [36]. 

Although the triple helix relies heavily on electrostatic interactions along the collagen fibrils, previous research has shown this process to also be sensitive to environmental factors such as pH or the ionic strength of the fiber formation buffer [36]. More specifically, at pH values that deviated from the isoelectric point of collagen molecules, fiber formation was slow or incomplete [38]. In the context of the extrudability of fibrillar collagen gel, previous work conducted by Lode et al. studied the effect of pH on the viscosity of the colloidal system. A pH of 4 was considered optimal, whereas an increase to pH 7 led to phase separation and loss of extrudability [38]. In the current study, pH was slightly lower than the optimal conditions established in the literature. Nevertheless, the combination of strong acidic regulation and mechanical grinding is presumed to have facilitated the homogenization of the collagen fiber dispersions and to have improved nozzle extrudability. In addition, ionic solutes such as the 135 mM sodium chloride (NaCl) used in a fiber formation buffer, are presumed to have increased the fibrillar nature of collagen, resulting in the ink being more fiber-like and extrudable whilst stabilizing collagen triple helices, as indicated in previous studies [36,39].

**Figure 1:**
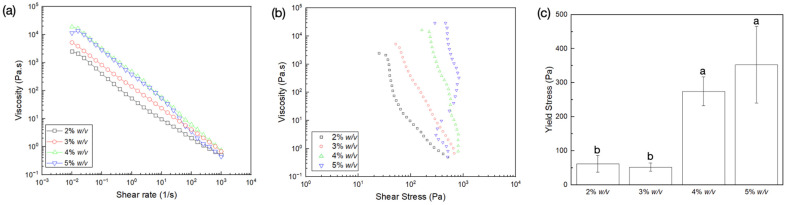
Flow sweep measurements of the various gel dispersions showing variation of viscosity (Pa.s) as a function of (**a**) shear rate (1/s) and (**b**) shear stress (Pa); (**c**) yield stress dependence on collagen dispersion concentration. Data presented as mean ± standard deviations. Letters represent statistically homogenous groups.

Flow sweep measurements demonstrated shear thinning behavior characterized by the reduction in viscosity as a function of shear rate (Figure 1a). At low shear rates, polymer chains demonstrated higher viscosity due to fibrillar entanglements [40]. As the shear rate increased, the entanglement of polymer chains could have reduced, causing a decrease in the overall effect of the material interaction and viscosity of the gels. Irrespective of collagen concentration, the absence of zero-shear viscosity followed by subsequent yielding of the collagen network in response to the applied shear indicated characteristic viscoplastic behavior. Viscosity versus shear stress plots (Figure 1b) helped determine the yield stress of the gels, characterized as the region at which viscosity showed a rapid decrease as a function of shear stress [41]. The increase in collagen concentration resulted in an increase in yield stress (Figure 1c). This was seen as vertical regions of stress on the viscosity versus shear stress plot immediately upon the application of shear stresses (at low shear rates ~0.01 1/s). While this phenomenon was apparent at all concentrations, the stress required to cause yielding increased with a rise in collagen concentration. This can primarily be attributed to the increase in interactions due to the proximity of collagen fibers at higher concentrations. While both 4% *w*/*v* and 5% *w*/*v* dispersions demonstrated similar yield stress (*p =* 0.656), they exhibited significantly higher strength when compared to 2% *w*/*v* (*p* = 0.038 and *p* = 0.012, respectively) and 3% *w*/*v* dispersions (*p* = 0.026 and *p* = 0.008, respectively). Importantly, the flow curve of the 5% *w/v* collagen dispersion deviated towards a region of reduced shear stress at higher shear rates (>25 1/s), which could be attributed to wall slipping. Nonetheless, the 5% *w*/*v* dispersion permitted homogeneous extrusion of the material through a nozzle orifice (not shown) and was hence used in all subsequent experiments.

**Figure 2:**
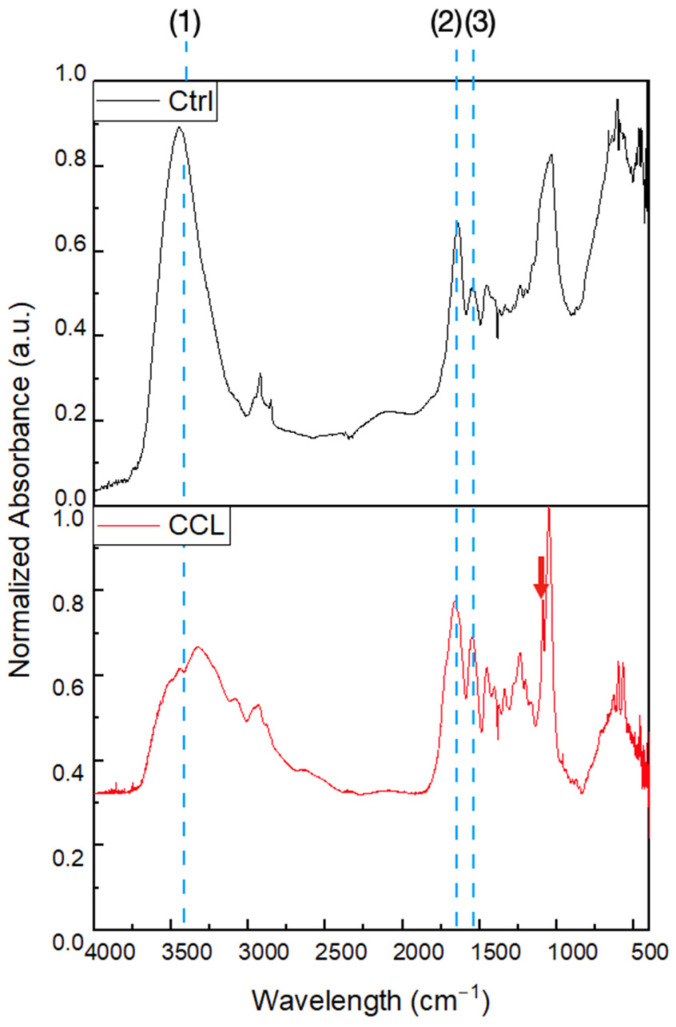
FTIR of the Ctrl and CCL groups. (1), (2), and (3) represent amide I, amide II, and amide III functional groups, respectively. Red arrow highlights ester band presence at 1080 cm^−1^ in the CCL group.

Pre- and post-processing conditions used in the synthesis of scaffolds unavoidably alter the physical, chemical, and biological properties of materials, which could hinder their pre-clinical or clinical applicability [42]. Fourier Transform Infrared (FTIR) spectra (Figure 2) of the control (Ctrl) and chemically crosslinked and lyophilized (CCL) groups demonstrated similar absorption spectra between the wavelengths examined. More specifically, both groups revealed the presence of absorbance bands corresponding to the hydroxyl group between 3230–3550 cm^−1^, amide I at 1690 cm^−1^ (highly characteristic of intermolecular hydrogen bonding), and amide II/amide III at 1680 cm^−1^. Importantly, spectra of CCL demonstrated an additional peak at 1080 cm^−1^, indicating the presence of an ester functional group. This was possibly caused by the formation of ester linkages from reactions between the carboxyl groups of EDC and amino and hydroxyl groups of the collagen dispersions [23]. As such, FTIR also demonstrated that the low temperatures and acidic pH that were maintained during post-processing steps were successful in preventing denaturation, as all other peaks of the Ctrl and CCL groups overlapped.

**Figure 3:**
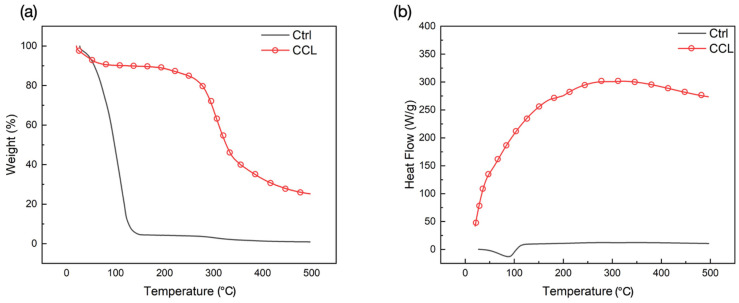
(**a**) TGA and (**b**) DSC spectra of the Ctrl and CCL groups (endotherm-up).

Thermogravimetric Analysis (TGA)/Differential Scanning Calorimetry (DSC) results further confirmed that crosslinking was achieved in the CCL group, indicated by its greater thermal stability in contrast to the Ctrl group prior to 100 °C (Figure 3a). TGA demonstrated two distinct stages of mass loss within the range of temperatures tested. Between room temperature (25 °C) and 150 °C, loss of water in both groups was seen as a drop in weight. On the other hand, loss in weight between 150–350 °C corresponded to the degradation of collagen and successive combustion of the residual organic components. Maximum weight loss was observed in the Ctrl group at the end of the test (Table 1). Corresponding DSC curves (Figure 3b) showed characteristic endothermic transitions in both groups, where the CCL group denatured at higher temperatures in comparison to the Ctrl group (Table 1). This higher denaturation temperature in the CCL group could be attributed to the presence of solvents or co-solvents (N-(3-Dimethylaminopropyl)-N′-ethylcarbodiimide hydrochloride (EDC), acetone, and water) used during the crosslinking and lyophilization processes. Previous studies have also indicated that approaches like lyophilization can help in fibrillar alignment, thereby increasing the denaturing temperature [43]. Additionally, crosslinked fibrillar structures that consist of Type I collagen have been reported to possess distinct structural features, including a higher degree of internal crystallinity, which also elucidate higher denaturation temperatures [44,45]. 

**Figure 4:**
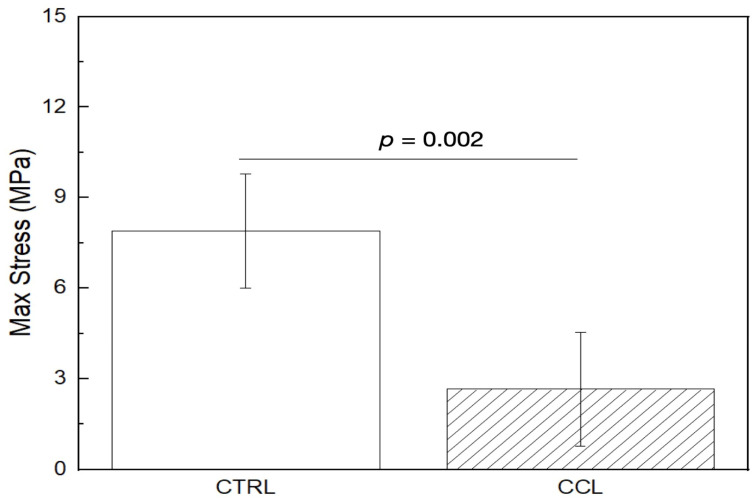
Maximum tensile stress (mean ± 95% CI) of the Ctrl and CCL groups. *p* < 0.05 is statistically significant.

Chemical crosslinking and lyophilization (CCL) revealed a statistical difference in maximum tensile stress (MPa) relative to untreated collagen scaffolds (Ctrl) (*p =* 0.002), with the Ctrl group exhibiting a higher resistance to deformation (Table 1 and Figure 4). While cross-linking of collagen with aldehydes like GA involves the formation of short aliphatic chains, cross-linking with EDC has been shown in the literature to result in the production of polypeptide chains that are directly coupled together [46]. However, contradictorily, results from previous studies have also shown that crosslinking results in intra-fiber bonding, as opposed to inter-fiber bonds, owing to gaps between successive chains [47]. In the context of the current evaluation, these gaps could have caused the early failure of brittle collagen fibers formed after extensive intra-fiber crosslinking, lowering the overall mechanical strength of the printed constructs. This warrants further studies that explore the combinatory effect of chemical and physical crosslinking which could potentially help mitigate the adverse effects of both aforementioned crosslinking methodologies.

**Figure 5:**
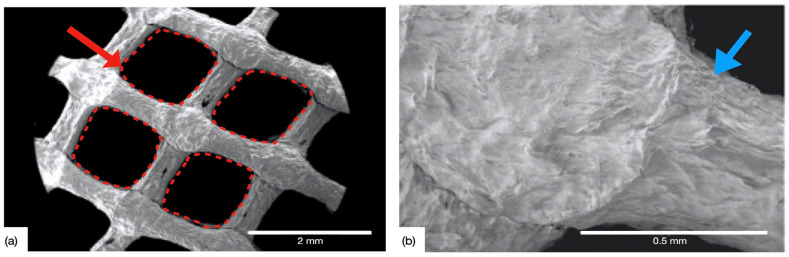
SEM of the 3D-printed CCL collagen scaffolds, (**a**) showing the engineered pore architecture (red arrow and dashed red boxes), and (**b**) high magnification micrograph depicting the intersection point of two successive layers, characterized by rod widening (blue arrow).

Figure 5a depicts the engineered pore architecture of the 3D-printed collagen scaffold. Upon observation under Scanning Electron Microscopy (SEM), the porous architecture was found to be intact following the post-processing steps (in the CCL group). Collagen dispersion exhibited shape retention capabilities as evidenced by the circular morphology of extruded rods. Figure 5b displays the intersection of rods at a higher magnification where a slight change in morphology (rod widening) was observed, owing to layer overlap. The 5% *w*/*v* dispersion also helped promote the multi-layer stacking capability of the gel during DIW and demonstrated the ability to be formed into spanning structures (lattices) [48]. 

DIW of colloidal gels for robocasting of spanning structures that undergo rapid drying-induced pseudoplastic to dilatant transition have been shown in previous studies to be incapable of fully supporting their own weight during assembly [20,21,49]. These difficulties associated with controlling drying kinetics during multilayer fabrication were not observed in the collagen gel used in this work, as it preserved its high-viscosity and glassy state after extrusion. Printed scaffolds were capable of supporting their self-weight after extrusion which allowed for the application of post-processing steps required to facilitate the shape fidelity of printed constructs. However, more robust time-sweep rheological analyses are required to monitor for changes in gel viscosity post-extrusion in follow-up studies.

**Figure 6:**
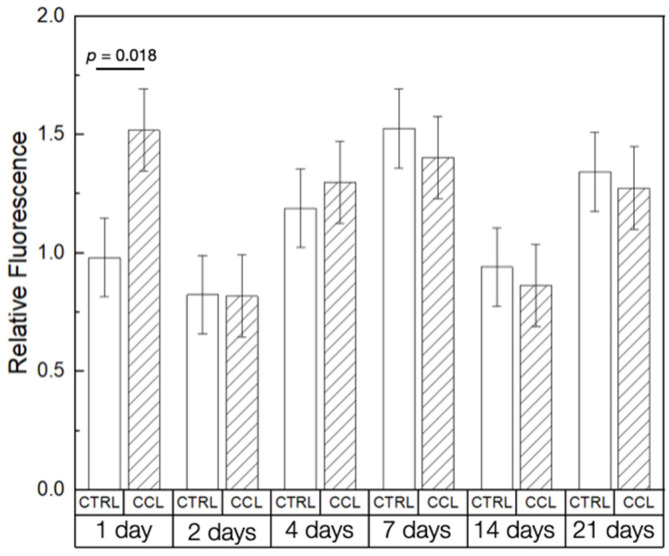
PB cell viability of the Ctrl and CCL groups at different time points. Data presented as means ± 95% CI. *p* < 0.05 is statistically significant.

The in vitro experiments confirmed the suitability of 3D-printed collagen scaffolds to serve as tissue engineering scaffolds. Presto Blue (PB) cellular viability assays showed with no adverse effect of post-processing steps (crosslinking and lyophilization) on the scaffolds’ biocompatibility at the time points evaluated (Figure 6). The Ctrl and CCL groups demonstrated a significant difference in viability at the 1-day time point, immediately following cell seeding (*p* = 0.018). At all other time points evaluated, cell viability was statistically homogenous between the groups, with a net increase in viability between days 2 and 7, owing to cellular proliferation. Cellular viability reduced in succeeding time points, possibly due to the ductile deformation of the scaffolds due to their submersion in culture media [50]. This is in agreement with previous studies which have indicated that the structural stability of scaffolds dictates cellular behavior and viability [51]. Between days 14 and 21, differentiation and successive calcification of human osteoprogenitor (hOP) cells on the surface of the scaffolds could have resulted in an increase in structural stability leading to an increase in relative fluorescence [52]. The analysis presented in the current investigation does not serve as conclusive proof of this phenomenon but necessitates follow-up studies to determine the effect of calcification on the strength of 3D-printed collagen scaffolds. 

**Figure 7:**
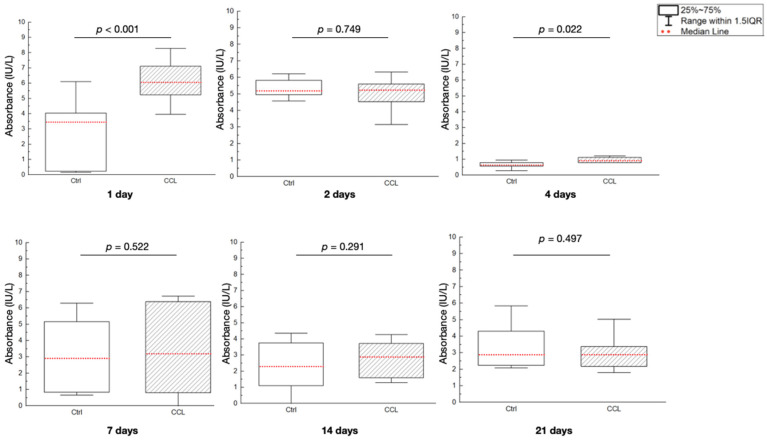
ALP gene expression, expressed as absorbance (IU/L) of the Ctrl and CCL groups at the various time points. Data presented as medians (dashed red lines) and corresponding interquartile ranges (IQR). *p* < 0.05 is statistically significant.

Alkaline phosphatase (ALP) activity (absorbance (IU/L)) was significantly different between the Ctrl and CCL groups at 1 and 4 days after seeding (*p* < 0.001 and *p* = 0.022, respectively), with the CCL group showing higher ALP expression (Figure 7). At all other time points evaluated, ALP activity was statistically homogenous between groups. In previous research, EDC crosslinked collagen was shown to increase the attachment of HT1080 cells five-fold, as crosslinked scaffolds were shown to remain stable in physiological conditions [36,53]. Moreover, studies have indicated that the peptization and crosslinking processes used to obtain colloidal, fibrillar collagen can further diminish antigenicity and improve biocompatibility [54,55]. Perhaps more importantly, crosslinked collagen has been shown to comprise adhesive ligands that regulate cellular response and are required by bone and soft-tissue forming cells to bind onto a substrate immediately following cell seeding—at the early evaluation time points [36,56]. This elucidates the cause for higher cellular viability (at the 1-day time point) and ALP expression (at the 1- and 4-day time points) in the CCL group relative to Ctrl.

## 3. Conclusions

Fibrillar collagen was synthesized from Type 1 bovine dermal collagen and successfully formulated into a colloidal gel for extrusion through a DIW printer. Crosslinked and lyophilized fibrillar collagen scaffolds were stable at 37 °C and presented adequate yield strength, which allowed for the printing of spanning lattice structures capable of supporting their own weight upon extrusion. 3D-printed scaffolds promoted cellular attachment and proliferation, making them a promising material for customized, patient-specific tissue-regenerative applications. However, despite its unique advantages to serve as scaffolds for tissue engineering, further characterization and biological evaluation are warranted. While the study provides a basis for the development of a 3D-printable, fibrillar collagen gel, successive studies are warranted to fully explore other post-processing techniques and in vivo biocompatibility through robust pre-clinical testing.

## 4. Materials and Methods

### 4.1. Collagen Gel Dispersion

Type I Bovine Collagen (bulk collagen) was procured from Viscofan (formerly Nitta Casings, Somerville, NJ, USA) and primarily comprised a triple helical structure with an average molecular weight of 407 kDa. Prior to synthesizing colloidal gels, bulk collagen was lyophilized for 72 h and subsequently ground into powder form. The ground collagen was converted into gel dispersions of varying weight percentages of collagen (2%, 3%, 4%, and 5% *w*/*v*) mixed with acetic acid (pH 2.4) in an overhead stirrer at 60 rpm at room temperature for 30 min. Following stirring, the resultant dispersions were filtered using a 140–200 μm stainless-steel mesh, after which they were subjected to centrifugation at 2400 rpm at 20 °C for 20 min. The resultant dispersions were stored at 4 °C prior to further evaluation.

### 4.2. Rheological Analysis

Gels were subjected to flow sweep measurements between a shear rate of 0.001 s^−1^ and 1000 s^−1^ (~35 s/step) varied logarithmically, on a rheometer (DHR-2, TA Instruments, New Castle, DE, USA). Gels were allowed to acclimate to room temperature and measurements were obtained with the gels loaded between 2 smooth stainless-steel parallel plates 20 mm in diameter and set 1.5 mm apart. Tests were carried out at 25 °C using a solvent trap cover to prevent drying of the gels.

### 4.3. 3D Printing of Collagen Gel Dispersions and Post-Processing

**Figure 8:**
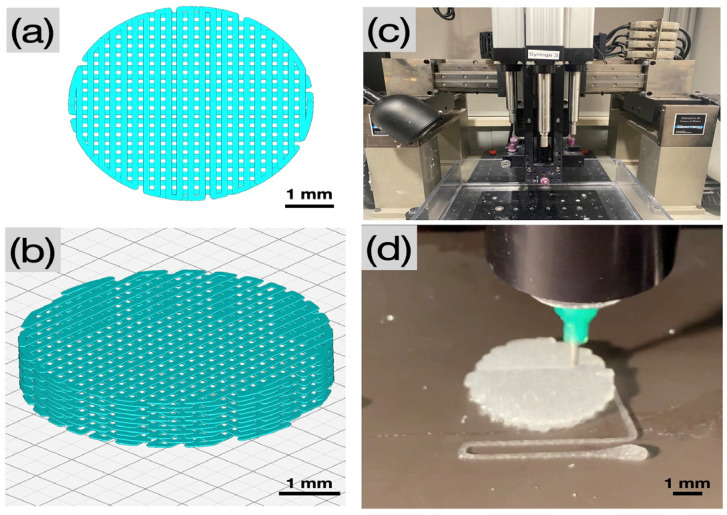
Pictographs of (**a**,**b**) the CAD of a cylindrical scaffold in top view and in isometric view, respectively, (**c**) the DIW printer, and (**d**) extrusion of 5% *w*/*v* collagen colloidal gel (dispersion) onto a dry substrate through a precision dispensing nozzle (Nordson, Westlake, OH, USA).

A custom computer-aided design (CAD) software, RoboCad 5.0 (3D Inks LLC, Tulsa, OK, USA), was used to design lattice-based cylindrical scaffolds 2 mm in thickness and 5 mm in diameter (Figure 8a,b). The collagen gel was loaded into 10 mL syringes (Nordson, Westlake, OH, USA) equipped with a 0.840 µm diameter (*d*) extrusion precision dispense tip (Nordson, Westlake, OH, USA). The material was extruded in a layer-by-layer fashion onto a flat, dry substrate using a custom-built DIW 3D printer (3D Inks LLC, Tulsa, OK, USA) (Figure 8c). Scaffolds were printed with speed (*v*) set at 2 mm/s, layer overlap (*O* = πd/4) set to 0.660 µm, and at an extrusion rate (*Q* = 0.25 π(*d*)^2^*v*) of 1.1 µL/s.

Immediately following printing (Figure 8d), the collagen scaffolds were placed in a fiber formation buffer solution (135 mM sodium chloride, 30 mM sodium phosphate dibasic heptahydrate, and 30 mM N-tris(hydroxymethyl) methyl-2-aminoethane sulfonic acid (TES)) for 24 h at 37 °C. The scaffolds were rinsed with isopropanol, followed by a subsequent rinse in distilled water, and were allowed to dry at room temperature for 12 h under quiescent conditions. Scaffolds were then subjected to the following treatments: (i) Ctrl—no post processing, and (ii) CCL—post-processing with chemical crosslinking and lyophilization. Post-processing in the CCL group included immersion of scaffolds in a solution of EDC in 90% acetone at room temperature for 24 h, rinsing in 50% acetone for 4 h followed by disodium hydrogen phosphate for 2 h. Contents were rinsed with distilled water and allowed to dry. After chemical crosslinking, scaffolds were lyophilized for 20 h.

### 4.4. Post-Print Characterization (Physico/Chemical and Mechanical Analysis)

FTIR (Magna 550-IR Series instrument (Magna, Waltham, MA, USA) analyses were conducted over the range of 4000 to 400 cm^−1^. Pellets were fabricated by mixing 1 mg of the desired specimen with 250 mg potassium bromide (KBr) to serve as the background. TGA and DSC were performed to analyze the behavior of the material subjected to thermal conditions. Measurements were obtained using a thermogravimetric analyzer (SDT Q600, TA Instruments, New Castle, DE, USA). Tests were carried out using ~10 mg of the sample at a linear ramp rate of 10 °C/min from 25 °C to 500 °C in the presence of a N_2_/O_2_ gas mixture.

SEM was used to obtain topographical information on the 3D-printed constructs. The samples were observed under a scanning electron microscope—Hitachi TM-4000 SEM (Hitachi High-Technologies Corporation, Tokyo, Japan)—at 5 kV. Mechanical (tensile) testing was performed using a Chatillon Force Measurement TCM 100S system (Ametek, Largo, FL, USA), using clamping grips (*n* = 5/treatment group). The machine was equipped with a ± 100 N load cell and the crosshead speed was set to 1 mm/min. All tensile testing followed ASTM D638-14 standard test methodology [57]. 

### 4.5. In Vitro Biocompatibility and Alkaline Phosphatase (ALP) Expression

hOP cells were harvested (NYU IRB approval: #S18-01579) and allowed to expand in culture media comprising Dulbecco’s Modified Eagle’s Medium (DMEM) supplemented with 10% Embryonic Stem-cell Fetal Bovine Serum (ESFBS), and 1% Antibiotic/Antimycotic (Thermo Fisher Scientific, Waltham, MA, USA). Cells were stored at 37 °C under 5% CO_2_ in a humidified incubator. After 2 days, cells were washed with Phosphate-buffered Saline (PBS) (Thermo Fisher Scientific, Waltham, MA, USA), and fresh media was supplemented. When ~70% confluence was achieved, cells were detached from the culture plate using trypsin (Thermo Fisher Scientific, Waltham, MA, USA), and counted using the automated cell counter Countess II FL (Life Technologies, Waltham, MA, USA). Prior to in vitro testing, cylindrical scaffolds were sterilized using an X-Ray irradiator (CellRad X-Ray Irradiator, Faxitron, Tucson, AZ, USA) at 125 kV for 30 min.

Sterilized scaffolds were placed into a 48-well plate, following which hOP cells (~10^5^) were directly seeded onto the scaffolds. More specifically, cells were suspended in 100 µL of culture media and introduced directly upon the surface of the scaffolds. Following cell seeding, each well was topped with 200 µL of culture media to ensure complete submersion of the scaffold before being placed in the incubator. PB Cell Viability Reagent (Thermo Fisher Scientific, Waltham, MA, USA) was used to evaluate the viability and proliferation of cells on the collagen scaffolds 1, 2, 4, 7, 14, and 21 days after seeding (*n* = 6/group). Fluorescence measurements were recorded using a plate reader (FilterMax F5 Multi-mode Microplate Reader, Molecular Devices, San Jose, CA, USA). The fluorescence assay was conducted after an orbital shake for 5 s. An excitation wavelength and an emission wavelength of 535 nm and 595 nm were utilized, respectively. Fluorescence values of all groups, and at every time point, were normalized by the fluorescence value obtained for the Ctrl group at the 1-day time point to produce a time-series effect (expressed as relative fluorescence).

ALP levels were quantified after 1, 2, 4, 7, 14, and 21 days. Each well plate was filled with DMEM (50 μL) from each time point evaluated and received 150 μL of working solution (200 mM magnesium acetate +1000 mM p-NPP buffer solution) (BioAssay Systems, Hayward, CA, USA). Absorbance values (IU/L)) were obtained at a wavelength of 405 nm (FliterMax 5, Molecular Devices, Sane Jose, CA, USA). One reading was obtained shortly after mixing the media and working solutions, whereas a second reading was taken after 4 min. ALP activity (absorbance) was calculated using the following equation:

$$ALP\;Activity\;\left(absorbance\right)\;\left(\frac{IU}{L}\right)\;=\;\frac{(OD\;sample\;at\;4\;\min\;-\;OD\;sample\;at\;0\;\min)\;\times \;35.3}{\left(OD\;calibrator\;-\;OD\;H_{2}O\right)}$$

### 4.6. Statistical Analysis

All descriptive data are depicted in graphs as mean values with corresponding 95% confidence intervals (mean ± 95% CI). One-way analyses of variance (ANOVA) were performed on IBM SPSS (IBM, Armonk, NY, USA) and *p* ≤ 0.05 was considered statistically significant. If the distribution was not normal, an equivalent non-parametric test (Kruskal–Wallis) was utilized for significance at *p* < 0.05, and data presented as median and respective interquartile ranges (median (IQR)) or as mean values with corresponding standard deviations (mean ± SD). 

## Figures and Tables

**Table 1:** Thermal and mechanical properties of the different groups used in this study.

Experimental Group	% Weight Loss (between 25 °C and 150 °C)	% Weight Loss (between 150 °C and 350 °C)	Endothermic Denaturation Temperature (°C)	Maximum Tensile Stress (MPa) (Mean ± 95% CI)
Ctrl	95.43	2.66	85.69	7.89 ± 1.89
CCL	10.31	48.48	114.72	2.65 ± 1.89

## Data Availability

The data that support the findings of this study are available on request from the corresponding author.
